# The complete mitochondrial genome of *Enicospilus ramidulus* (Linnaeus, 1758) (Hymenoptera: Ichneumonidae)

**DOI:** 10.1080/23802359.2026.2632461

**Published:** 2026-03-04

**Authors:** Yingzheng Tian, Sarina, Haiying Huang, Rika Namu, Bingshuang Qin, Xiaoxiao Song, Guoquan Wang, Ning Wang

**Affiliations:** a College of Agriculture, Guangxi University, Nanning, China; b Administration Bureau of Inner Mongolia, Xilingol Grassland National Nature Reserve, Xilingol League, China; c Grassland Technical Extension Station of Xilingol League, Xilingol League, China; d Xinjiang Agricultural University, Urumqi, China; e Chinese Academy of Agricultural Sciences Grassland Research Institute, Hohhot, China

**Keywords:** *Enicospilus ramidulus*, mitochondrial genome, phylogenomics, Ophioninae

## Abstract

*Enicospilus ramidulus* (Hymenoptera: Ichneumonidae) is a common parasitoid of Noctuidae larvae, with potential role in natural pest control. Here, we sequenced and annotated the complete mitochondrial genome of *E. ramidulus*. The mitochondrial genome spans 15,424 bp with an A + T content of 83.7% It comprises 13 protein-coding genes (PCGs), 22 tRNAs, two rRNAs, and a control region (CR). Phylogenetic analyses conducted using the maximum-likelihood method supported a close relationship between *E. ramidulus* and *E. sp.* within the subfamily Ophioninae. This study enhances our understanding of the mitochondrial genome structure of *E. ramidulus*, and contributes to the broader research field of Ichneumonidae genomics.

## 1. Introduction

Ichneumonidae is the largest Hymenoptera family (comprises approximately 48,000 described species) (Wei et al. 2010; Peters et al. 2017;  Sharanowski et al. 2021), constituting over 20% of all described Hymenoptera species (Veijalainen et al. 2013). This family exhibits ovipositional behaviors targeting host larval, pupal, and adult stages, ultimately causing host mortality for sustenance (Quicke 2015). Ichneumonids parasitize diverse holometabolous insects, particularly Coleoptera and Lepidoptera, with some lineages associating with spiders (Broad et al. 2019). They play an important role in maintaining natural balance and contributing to pest biocontrol (Steven et al. 2015). However, their mitochondrial genomes remain understudied, with only 23 partial mitochondrial gene fragments of *Enicospilus ramidulus* (Linnaeus, 1758) have been released on NCBI (as of 14 July 2025). This gap in knowledge highlights the need for further research into the mitochondrial genomics of this family.

*Enicospilus ramidulus* (Linnaeus, 1758) is a parasitic wasp belonging to the subfamily Ophioninae (Hymenoptera: Ichneumonidae). Notable for its nocturnal behavior and its role as a parasitoid of Lepidoptera larvae (Gauld 1988a, 1988b; Broad and Shaw 2016). It is distributed across the Afrotropical, trans-Palaearctic and Oriental regions (Yu et al. 2016). In Europe, it has been recorded from countries including the United Kingdom, Germany, Sweden, and Italy (Broad and Shaw 2016). In Northeast Asia, its presence is well-documented in Japan (throughout the archipelago from Hokkaido to Kyushu) (Shimizu et al. 2020). *E. ramidulus* can be identified by several key morphological features: the presence of two discrete, pigmented sclerites in the forewing’s discosubmarginal cell, a uniformly testaceous mesosoma lacking dark patches, and a metasoma that abruptly transitions to black starting from the 5th or 6th tergite onwards (Johansson 2018; Ihsan et al. 2022). Additionally, its long antennae (54–60 flagellar segments) further distinguish *E. ramidulus* within the genus *Enicospilus* (Gauld 1988a, 1988b).

Mitochondrial genomes are valuable for phylogenetic studies due to small size, maternal inheritance, and high mutation rates (Boore 1999), serving as effective molecular markers for population genetics and the evolutionary history of various species (Huang et al. 2016). Ichneumonidae mitogenomes are characterized by high A + T content, gene rearrangements, and conserved gene order (Dowton et al. 2003; Zheng et al. 2022a, 2022b). This study presents the first complete mitochondrial genome sequence of *E. ramidulus*, establishing a foundation for future phylogenetic and systematic research on this species.

## 2. Materials and methods

### 2.1. Sample collection and preservation

**Figure 1.**
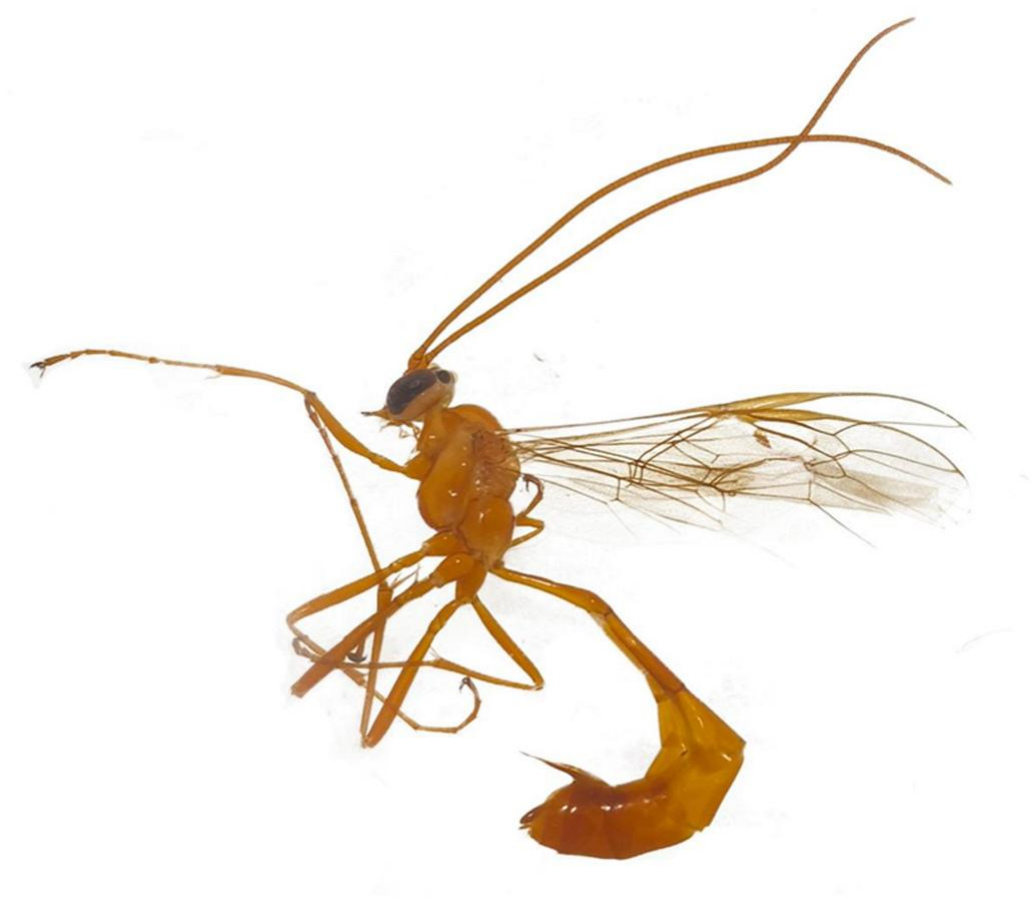
Adult image of the *Enicospilus ramidulus* (Linnaeus, 1758). The photo of species was taken by the first author Xiaoxiao Song using canon EOS 77D (canon, Tokyo, Japan) in the Institute of Grassland research of CAAS, Inner Mongolia, China.

A male specimen of *E. ramidulus* was collected from the Gonger Gacha, Darihan Ula Sumu, Hexigten Banner, Inner Mongolia, China (latitude 43°26′11.232″N, longitude 116°47′55.002″E) during 11–18 July 2021, using a malaise trap. The specimen was collected and identified by Xiaoxiao Song based on morphological characteristics and COI barcoding. The specimen was morphologically identified as Enicospilus ramidulus based on the following key characteristics: body length 15.0 mm; forewing length 11.5 mm; antenna with 53 flagellomeres; mesosoma uniformly testaceous; metasoma turning fuscous from approximately the 5th tergite onward; and the presence of two pigmented sclerites in the forewing discosubmarginal cell. The specimen is preserved in 95% ethanol and has been deposited in the Entomological Museum of the Institute of Grassland Research of CAAS (specimen number IGR600177, contact: Ning Wang: wangningis@163.com) (Figure 1). All collection and experimental procedures were approved by the Administration Committee of Experimental Animals of Inner Mongolia Province and the Ethics Committee of CAAS.

### 2.2. DNA extraction and sequencing

The DNA sample was sent to Biomarker Technologies Co. (Beijing, China) for DNA extraction and genome sequencing. DNA extraction was conducted following their standardized CTAB-based protocol, which included tissue homogenization in liquid nitrogen, lysis with CTAB buffer, purification using chloroform–isoamyl alcohol, and magnetic bead-based DNA purification. Subsequently, the sample was processed to construct a sequencing library for the Illumina NovaSeq 6000 platform (San Diego, CA). Quality control and assembly were performed using MitoZ v2.3 (Meng et al. 2019). The filtered reads, totaling 4.43 GB, yielded an average sequence coverage depth of 1918× (Figure S1).

**Figure 2.**
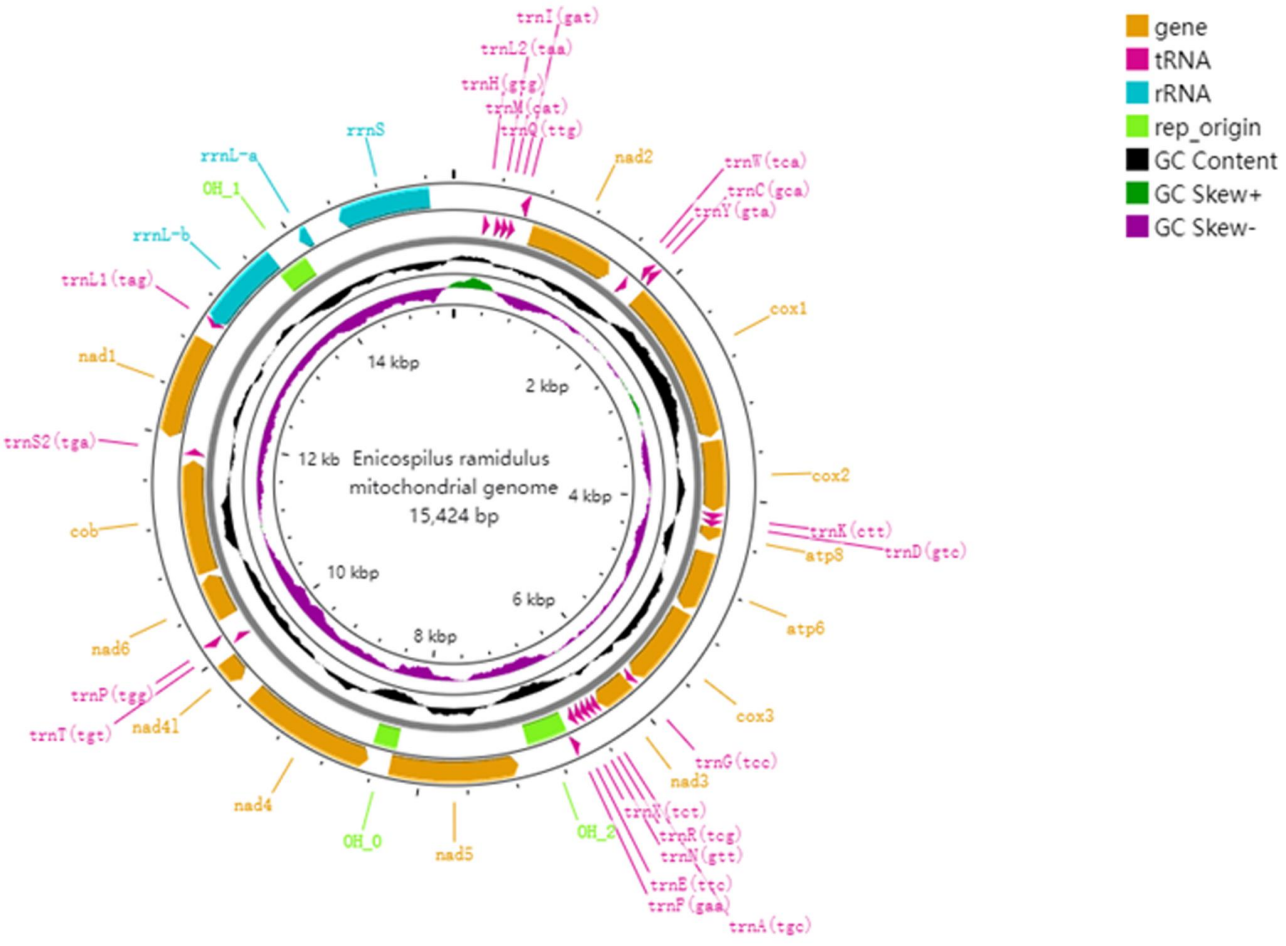
Genome map of *E. ramidulus.* The circular mitochondrial DNA is depicted as a double-stranded structure, with the H-strand on the outer ring and the L-strand on the inner ring. Different colors indicate different types of genes and regions.

The assembled mitochondrial genome fragment was initially examined to confirm its circular structure before conducting annotation on the MITOS web server (Bernt et al. 2013). The mitochondrial annotation was validated through manual curation with MEGA v7.0 (Kumar et al. 2016), where the identified genes were aligned with homologous mitochondrial sequences of *E. ramidulus* from published datasets. The mitochondrial genome sequence and gene annotations were submitted to the National Center for Biotechnology Information GenBank database under accession number PQ221458. The complete mitochondrial genome was visualized using the CGView online server (https://proksee.ca/) (Figure 2).

### 2.3. Phylogenetic analysis

**Figure 3.**
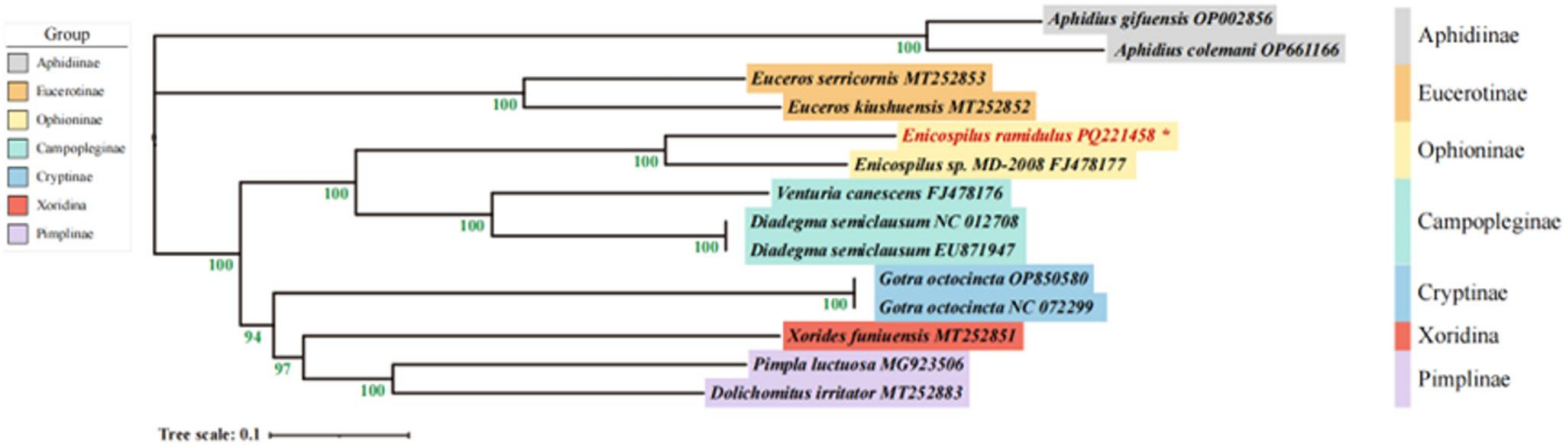
The maximum-likelihood (ML) phylogenetic tree based on the mitochondrial sequences of 14 species. Branches are scaled to genetic distance, with the scale bar indicating 0.1 substitutions per site. Values at nodes represent bootstrap support. * represents the newly sequenced mitochondrial genome in this study and it is also highlighted in red. All species involved in the tree have scientific names with accession number on right side. The complete mitochondrial sequences and accession ID were used as follows: *Aphidius gifuensis* OP002856; *Aphidius colemani* OP661166; *Diadegma semiclausum* NC012708, *Diadegma semiclausum* EU871947 (Wei et al. 2009); *Venturia canescens* FJ478176, *Enicospilus sp* FJ478177 (Dowton et al. 2009); *Xorides funiuensis* MT252851, *Euceros kiushuensis* MT252852, *Euceros serricornis* MT252853 (Zheng et al. 2022a, 2022b); *Gotra octocincta* NC_072299; *Gotra octocincta* OP850580; *Pimpla luctuosa* MG923506 (Tang et al. 2019); *Dolichomitus irritator* MT252883.

To investigate the phylogenetic relationship of *E. ramidulus*, we integrated the mitochondrial gene sequence with those of 13 other species data from NCBI. This comparison included 11 representatives from Ichneumonidae (three Campopleginae, two Pimplinae, two Cryptinae, two Eucerotinae, one Xoridinae, and one Ophioninae) and two outgroup species (*Aphidius gifuensis* Ashmead, 1906 and *Aphidius colemani* Viereck, 1912 from Braconidae) (Supplementary Table S1). The nucleotide sequences mentioned above were aligned using the ClustalW module in MEGA v11.0 with default parameters. The evolutionary history was inferred using the maximum-likelihood method with the GTR + I + G model, applying 1000 bootstraps replicates in MEGA v11.0 (Nei et al. 2000). The resulting tree with the highest log likelihood is presented in Figure 3. The visual representation of the tree was enhanced using ChiPlot (https://www.chiplot.online/). A version of the phylogenetic tree with branch lengths scaled to genetic distance is provided in the Supplementary Material (Figure S2).

## 3. Results

### 3.1. Mitogenome organization

The complete mitogenome sequence is 15,424 bp (Figure 2) and exhibits a typical circular structure. This genome contains 37 genes, including 13 protein-coding genes (PCGs), 22 tRNAs, two rRNAs, and a control region (CR). The nucleotide composition is predominantly composed of adenine (A) and thymine (T), which together account for 83.7% of the total nucleotide content (A = 42.3%, T = 41.4%). This A + T bias is consistent with the high levels observed in Hymenoptera mitochondrial genomes (Dowton and Austin 1997). The remaining nucleotide composition includes cytosine (C) at 9.7%, and guanine (G) at 6.5%. Besides, the sequence exhibits a negative GC-skew on the sense strand, while the antisense strand shows a positive GC-skew, consistent with asymmetric gene distribution across the two strands (Figure 2 and Table S2).

Comparative analysis with congeneric species revealed significant differences. The mitochondrial genome of *E. ramidulus* (15,424 bp) is 124 bp longer than that of *E. sp*. (15,300 bp) (Zheng et al. 2022a, 2022b). In terms of nucleotide composition, *E. ramidulus* exhibits an A + T content of 83.7%, while *E. sp*. shows a slightly higher A + T content of 85.1% (based on Wei et al. 2010). Both values reflect the characteristically high A + T richness typical of the Ophioninae subfamily (Wei et al. 2010).

The 13 PCGs in the mitochondrial genome span 11,325 bp (accounting for 73.4% of the total genome). Most PCGs initiate with ATN codons: eight genes (ND2, COX2, ATP8, ATP6, ND3, ND6, CYTB, and ND1) start with standard ATG codon, while three genes (ND4, ND4L, and ND5) start with ATT. The start codon for COX1 could not be definitively determined through automated annotation and was therefore left unspecified in the final annotation. Among the PCGs, 10 terminate with the canonical stop codon TAA, while three (ND2, ND3, and cytb) end with incomplete single T codons. The 22 tRNAs vary in length from 54 to 77 bp, and predominantly adopt cloverleaf secondary structures. However, the tRNA for alanine (trnA) is an exception, as it lacks the dihydrouridine (DHU) arm.

### 3.2. Phylogenetic analysis

Phylogenetic analysis was conducted using maximum-likelihood methods based on the General Time Reversible + G + I model, incorporating mitochondrial genomes from 12 species of Ichneumonidae and two outgroups species from Braconidae (*Aphidius gifuensis* and *A. colemani*). The values indicated on the nodes represent bootstrap support from maximum-likelihood analysis. The results demonstrate that the ingroup clustered into five distinct branches. Notably, the analysis revealed a close relationship between the subfamilies Ophioninae and Campopleginae, indicating that they share a common evolutionary origin from a common ancestor. Within the subfamily Ophioninae, *E. ramidulus* was found to be closely related to *E. sp*. This relationship is strongly supported by a high bootstrap value (100). Additionally, the subfamily Aphidiinae, represented by *A. colemani* and *A. gifuensis*, forms a distinct lineage that is separate from Eucerotinae, which aligns with their specialized role in aphid parasitism.

## 4. Discussion and conclusions

This study presents the first complete mitochondrial genome of *E. ramidulus.* While the arrangement of its 13 protein-coding and two ribosomal RNA genes conforms to the inferred ancestral hymenopteran gene order, it exhibits several derived tRNA gene rearrangements that are characteristic of the subfamily Ophioninae (Dowton et al. 2009) (Table S3). The COX1 gene presented annotation challenges, as its start codon could not be definitively determined, a situation occasionally encountered in hymenopteran mitochondrial annotations (Dowton et al. 2003). Phylogenetic analysis places *E. ramidulus* within the subfamily Ophioninae, where it clusters with its congener *E. sp.*, supported by high bootstrap values. This finding aligns with previous molecular studies that confirm the monophyly of the genus *Enicospilus* within the Ophioninae, which are characterized by their nocturnal behavior and parasitism of Lepidoptera larvae (Quicke et al. 2011). This work enriches Ichneumonidae mitochondrial genomic data and provides a foundation for future genetic research.

## Supplementary Material

Supplemental Material

Supplemental Material

## Data Availability

The genome sequence data that support the findings of this study are openly available in GenBank of NCBI (https://www.ncbi.nlm.nih.gov/) under the reference number PQ221458. The associated BioProject, BioSample, and SRA numbers are PRJNA1131919, SAMN42328548, SUB14530994, respectively.
