# Supplementary figures and images for: The complete mitochondrial genome of Enicospilus ramidulus (Linnaeus, 1758) (Hymenoptera: Ichneumonidae)
Source: Mitochondrial DNA B Resour. 2026 Mar 4;11(4):493–7. doi: 10.1080/23802359.2026.2632461 (PMC12961704; doi:10.1080/23802359.2026.2632461)

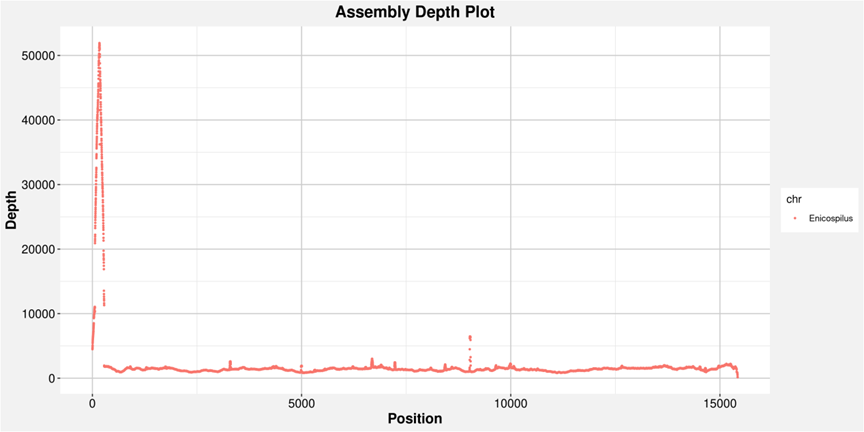

Supplement: Supplemental Material [file TMDN_A_2632461_SM1666.tiff]

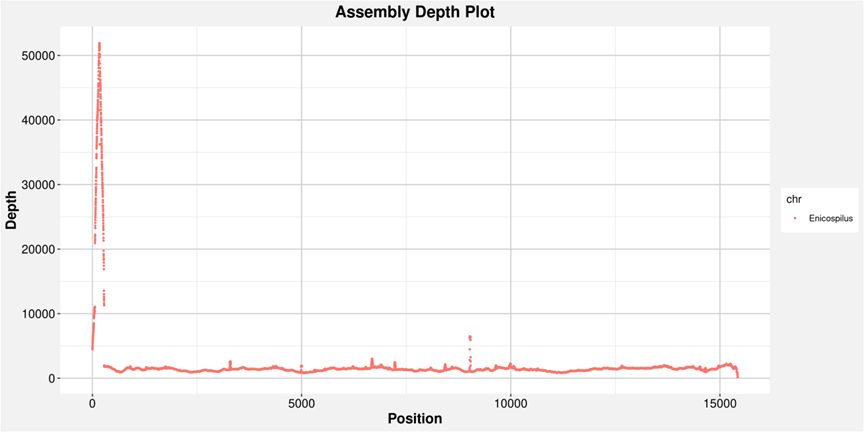

Supplement: Supplemental Material [file TMDN_A_2632461_SM1662.tif]
